# Pulmonary exposure to renewable diesel exhaust particles alters protein expression and toxicity profiles in bronchoalveolar lavage fluid and plasma of mice

**DOI:** 10.1007/s00204-024-03915-y

**Published:** 2024-12-29

**Authors:** Sarah McCarrick, Vilhelm Malmborg, Louise Gren, Pernille Høgh Danielsen, Martin Tunér, Lena Palmberg, Karin Broberg, Joakim Pagels, Ulla Vogel, Anda R. Gliga

**Affiliations:** 1 Institute of Environmental Medicine, Karolinska Institutet, Stockholm, Sweden; 2 Division of Ergonomics and Aerosol Technology, Lund University, Lund, Sweden; 3 Division of Combustion Engines, Lund University, Lund, Sweden; 4 National Research Centre for the Working Environment, Copenhagen, Denmark

**Keywords:** Diesel exhaust particles, Renewable fuels, Lung inflammation, Systemic effects, Cytokines, Intratracheal instillation, Protein profile

## Abstract

Exposure to diesel exhaust is associated with increased risk of cardiovascular and lung disease. Substituting petroleum diesel with renewable diesel can alter emission properties but the potential health effects remain unclear. This study aimed to explore toxicity and underlying mechanisms of diesel exhaust from renewable fuels. Using proximity extension assay (Olink), 92 proteins linked to inflammation, cardiovascular function, and cancer were analyzed in bronchoalveolar lavage fluid (BALF) and plasma in mice 1 day after pulmonary exposure to exhaust particles at doses of 6, 18, and 54 µg/mouse. Particles were generated from combustion of renewable (rapeseed methyl ester, RME13, hydrogen-treated vegetable oil, HVO13; both at 13% O_2_ engine intake) and petroleum diesel (MK1 ultra-low-sulfur diesel at 13% and 17% O_2_ intake; DEP13 and DEP17). We identified positive dose–response relationships between exposure and proteins in BALF using linear models: 33 proteins for HVO13, 24 for DEP17, 22 for DEP13, and 12 for RME13 (*p* value < 0.05). In BALF, 11 proteins indicating cytokine signaling and inflammation (CCL2, CXCL1, CCL3L3, CSF2, IL1A, CCL20, TPP1, GDNF, LGMN, ITGB6, PDGFB) were common for all exposures. Several proteins in BALF (*e.g.,* CCL2, CXCL1, CCL3L3, CSF2, IL1A) correlated (*r*_s_ ≥ 0.5) with neutrophil cell count and DNA damage in BAL cells. Interestingly, plasma protein profiles were only affected by RME13 and, to lesser extent, by DEP13. Overall, we identified inflammation-related changes in the BALF as a common toxic mechanism for the combustion particles. Our protein-based approach enables sensitive detection of inflammatory protein changes across different matrices enhancing understanding of exhaust particle toxicity.

**Supplementary Information:**

The online version contains supplementary material available at 10.1007/s00204-024-03915-y.

## Introduction

Diesel engine exhaust is a classified human carcinogen (IARC 2014) and has been associated with increased risk of lung and cardiovascular disease (Wilson et al. 2018; Long and Carlsten 2022; Vermeulen et al. 2014; Ge et al. 2020). In recent time, there has been a widespread promotion of renewable diesel fuels to replace conventional petroleum diesel with the potential to reduce net CO_2_ emissions and at the same time decrease particulate matter (PM) emissions (Othman et al. 2017; Bae and Kim 2017; Godri Pollitt et al. 2019; Agarwal et al. 2015). Renewable diesel fuels are derived from sources such as vegetable oils, animal fats, and waste products and can be utilized in traditional engines as a complete substitute or in blends. Rapeseed methyl ester (RME) is a first-generation renewable fuel, which is categorized as fatty acid methyl ester (FAME) fuel and characterized by an elevated oxygen fuel content (approximately 10%). Second-generation renewable diesel fuels feature hydrogen treatment to eliminate the oxygen content and result in a fuel more similar to petroleum diesel. This includes hydrogen-treated vegetable oil (HVO), a synthetic/paraffinic diesel produced from plant and animal sources that is chemically similar to petroleum diesel, but lacks aromatic content and has shorter and more uniform carbon chains (Aatola et al. 2009). Yet, despite the many benefits of these renewable diesel fuels, there is limited knowledge regarding health effects associated with their emissions (Møller et al. 2020). One human experimental study using healthy volunteers exposed for 3 h to HVO exhaust did not find signs of altered lung function, but did identify an increase in self-rated mild irritation of eyes and throat (Gren et al. 2022).

Inflammation is regarded as a key process in the development of health effects related to diesel exhaust particles (DEPs) (Ristovski et al. 2012; Steiner et al. 2016). Several biomarkers, particularly those linked to inflammation, oxidative stress, and DNA damage, have been proposed to predict respiratory effects attributed to exposure to particulate matter (Guo et al. 2022; Halappanavar et al. 2020; Nymark et al. 2021). Some commonly investigated biofluids include bronchoalveolar lung fluid (BALF) and plasma. The protein expression profile of BALF directly reflects the local biological changes in the airspace milieu, whereas plasma, a major part of the circulatory system, reflects more systemic effects. Acute phase response proteins such as C-reactive protein (CRP) and serum amyloid A (SAA) have been suggested as useful biomarkers for analyzing the risk of exposure to various particles including DEPs and nanomaterials (Higashisaka et al. 2011; Saber et al. 2014; Gutierrez et al. 2023; Hadrup et al. 2020). For example, pulmonary inflammation assessed as neutrophil influx has been shown to correlate to both pulmonary *Saa3* mRNA and plasma SAA3 protein levels, in response to various nanomaterials such as metal oxide nanoparticles and carbon nanotubes (Gutierrez et al. 2023; Saber et al. 2013; Poulsen et al. 2017; Danielsen et al. 2024).

In a previous study (Bendtsen et al. (2020)), the histopathological, inflammatory and genotoxic effects of pulmonary exposure to diesel exhaust particles (DEP) were investigated in female C57BL/6Tac mice at 1, 28, and 96 days after a single instillation exposure. The DEPs were generated from petroleum and renewable diesel (RME and HVO), respectively, by a modern heavy-duty diesel engine in a controlled laboratory environment (Gren et al. 2020). Particles derived from HVO and petroleum diesel induced dose-dependent inflammation in the lung measured as increased neutrophil and lymphocyte influx as well as acute phase response (higher *Saa3* level in lung) 1-day post-exposure. RME-derived particles were found to induce the least toxic response. The overall aim of this study was to further explore the toxicity and underlying mechanisms-of-action related to different types of renewable DEPs. This was done by building on the study of Bendtsen et al. (2020), investigating acute changes (24 h) in the protein profile of 92 proteins related to inflammation, cardiovascular function, and cancer in BALF and plasma from mice after instillation exposure. Further, this study sought to identify potential early and sensitive protein biomarkers in BALF and plasma that are predictive of lung toxicity after acute pulmonary exposure to various DEPs. Ultimately, this could be used to predict and compare the potential toxicity of DEPs or other types of combustion particles.

## Methods

### Particle collection

Particles were generated, collected, and characterized as previously described in Gren et al. (2020). Briefly, particles were generated with a modern heavy-duty diesel engine being operated on either petroleum diesel (Swedish ultra-low sulfur MK1) or two types of renewable diesels: hydrogen-treated vegetable oil (HVO; no aromatics and no oxygen content) and rapeseed methyl ester (RME, no aromatics but 10.6% oxygen) with no external exhaust after treatment system. The engine was operated at a constant low load but with a variation in the amount of exhaust gas recirculation (EGR), which is a commonly used technique to reduce combustion temperatures to decrease NOx emissions from engines. Simultaneously this increases soot and PAH formation. The amount of EGR changes the intake O_2_ concentration to the combustion cylinder, which in turn changes combustion temperature, and thus the combustion conditions. In this study, petroleum diesel at ~ 13% intake O_2_ (NO_x_ eliminating EGR levels) and ~ 17% intake O_2_ (low EGR, minor NO_x_ reduction effect) were included and denoted as DEP13 and DEP17, respectively. The renewable diesel fuels (RME13 and HVO13) were sampled at ~ 13% intake O_2_. The study design facilitated a comparison between emissions from renewable fuels, HVO13, RME13, and DEP13 generated under the same engine conditions at 13% intake air O_2_ concentration. This condition also increased soot emissions which allowed for collection of generous amount of particles for toxicity testing. For comparison to more realistic engine operating conditions, we also included DEP17, particles collected from the engine operating at a lower amount of EGR and 17% intake air O_2_. Printex90 nanoparticles, a carbon black was used as reference particle.

Particles were collected on PTFE filters (Whatman PTFE, 150 mm, pore size 5 μm) using the final filter stage of a high-volume cascade impactor (HVCI, BGI Inc.). The last impactor stage provided a cutoff size of 1 μm (PM1) for the filter sampling. Particle characteristics as previously reported in Gren et al. (2020) and Bendtsen et al. (2020) are summarized in Table 1.

**Table 1:** Summary of particle characteristics as modified from Gren et al. (2020) and Bendtsen et al. (2020)

	RME13	HVO13	DEP13	DEP17	Printex 90
Average emitted PM1 (mg/m^3^) ^a^	34	34	96	3	–
Average emitted PM1 (mg/kWh) ^b^	144 ± 27	133 ± 15	386 ± 61	8 ± 2	–
% O_2_ in fuel ^a^	10	0	0	0	–
Intake O_2_% ^a^	13	13	13	17	–
Primary particle diameter (nm) ^a^	15 [13.7, 16.4]	20.9 [19.1, 22.8]	17.4 [16.2, 18.8]	16.1 [14.9, 17.5]	14.5 [13.7, 15.4]
Aggregate size (nm) ^b^	70 ± 3	90 ± 5	104 ± 8	62 ± 4	–
Estimated SSA (m^2^/g) ^b^	222 [203, 243]	160 [146, 174]	191 [177, 206]	207 [191, 224]	230 [217, 243]
Elemental to total carbon (EC/TC) ^a^	0.68	0.72	0.88	0.6	–
Organic to total carbon (OC/TC) ^a^	0.32	0.28	0.12	0.4	–
Total PAHs (μg/g) ^a^	2060	13,400	4599	1390	–
BaPeq (μg/g) ^a^	60	1067	165	59	–
Total all metals (μg/g) ^b^	2530	1990	905	15,500	–
Cr (μg/g) ^a^	7	11	7	52	ND ^c^
Mn (μg/g) ^a^	43	39	53	ND	1/0 ^c^
Fe (μg/g) ^a^	116	247	137	2115	9/12 ^c^
Cu (μg/g) ^a^	2291	1632	629	13,160	10/1 ^c^
Sr (μg/g) ^a^	37	41	54	ND	1/1 ^c^
ROS (fluorescence/μg)^b^	14,457	19,998	14,682	24,039	41,554
ROS (fluorescence/cm^2^)^b^	6512	12,499	7687	11,613	18,067

Data sourced from Bendtsen et al. (2020) is marked with (a) and data sourced from Gren et al. 2020 is marked with (b). For CB Printex90 metal, data are sourced from Bendtsen et al., (2019) and marked with (c); samples were analyzed in duplicates, separated by “/”

RME13 = rapeseed methyl ester at 13% O_2_ engine intake; HVO13 = hydrogen-treated vegetable oil at 13% O_2_ engine intake; DEP13 = MK1 ultra-low-sulfur diesel at 13% O_2_ engine intake; DEP17 = MK1 ultra-low-sulfur diesel at 17% O_2_ engine intake; ND = not detectable; SSA = specific surface area; PAHs = polycyclic aromatic hydrocarbons; PM1 = particulate matter < 1 μm, BaPeq = benzo[a]pyrene equivalent concentration for 12 PAH out of total of 63 measured PAHs and PAH derivatives; ROS = reactive oxygen species

### Mice

The study was in agreement with Directive 2010/63/EU of the European Parliament and of the Council of 22 September 2010 on the protection of mice used for scientific purposes, and the Danish Animal Experimentation Act (LBK 474 15/05/2014). The study was approved by The Animal Experiments Inspectorate under The Ministry of Environment and Food of Denmark (License: 2015-15-0201–00465) and the local Animal Welfare Committee responsible for ensuring implementation of 3R policy at the National Research Center for the Working Environment. For this sub-study, samples from 88 female C57BL/6Tac mice were used. Mice were 7 weeks old at arrival and group-housed in standard cages with 6–8 mice with ad libitum access to tap water and Altromin 1324 rodent diet. All mice were housed in 1290D euro standard Type 3 cages on saw-dust bedding with mouse house, wooden chew blocks and Enviro Dri nesting material as enrichment. The mice were kept at 21 ± 1 °C and 50 ± 10% humidity in a 12 h light–dark circle. The animal study was reported previously (Bendtsen et al. 2020).

### Animal exposure and sample collection

Particles were suspended in Nanopure Diamond Water with 0.1% Tween80. Nanopure Diamond Water with 0.1% Tween80 was prepared similarly for vehicle control. More details on the instillation procedure and sample harvest can be found in Bendtsen et al. (2020). Briefly, BALF and plasma were collected after 1-day post-instillation exposure to RME13, HVO13, DEP13, DEP17 and reference carbon black (CB) Printex90 particles. BALF and plasma samples from three dose levels (6, 18, 54 μg, corresponding to 0.31, 0.94 and 2.8 mg/kg bw, respectively) were included for DEP13, RME13, and HVO13 (*n* = 5–6), the two higher dose levels (18 and 54 μg) were included for DEP17 (*n* = 5), and one dose for CB (54 μg) (*n* = 6). Samples for vehicle control (*n* = 11) and extraction control (*n* = 6) were also included. Total number of animals was 88.

### Protein measurement

BALF and plasma samples were analyzed using proximity extension assay (PEA) technology (Olink Proteomics AB, Uppsala, Sweden; analysis performed at Affinity Proteomics, Scilife lab, Uppsala) for 92 unique proteins in the panel of Target 96 Mouse Exploratory. The selected proteins are related to the following main annotations of diseases and functions in Ingenuity Pathway Analysis (Qiagen): inflammatory response (*n* = 37), cell movement (*n* = 60), cardiovascular disease (*n* = 45) and cancer (*n* = 42). The analyzed proteins are listed in the supplementary material, Table S1. All samples in BALF and all samples except one in plasma (RME 18 μg) passed quality control and were included for analysis.

Protein levels were reported as normalized protein expression (NPX) values on a log2-scale. Proteins with more than 50% of the samples ≤ limit of detection (LOD) were excluded from the analysis, but not if at least one entire dose group in any exposure was above LOD, in which case the proteins were included. These criteria resulted in an exclusion of 42 proteins in BALF samples and 12 proteins in plasma (see Table S1 for excluded and included proteins). Finally, this resulted in 50 proteins in BALF and 80 proteins in plasma to be included in the statistical analysis.

### Data analysis

All analyses were performed using RStudio with R version 4.3.1. Venn diagrams were performed in https://www.biotools.fr/misc/venny.

#### Correlation between proteins in BALF and plasma and in vivo markers

Correlations between proteins in BALF, plasma, and in vivo toxic endpoints were evaluated using Spearman correlation by the *cor* function in R. In vivo toxicity markers included in the correlation analysis were numbers of cells in BAL (total, macrophages, lymphocytes, neutrophils, eosinophils, epithelial), genotoxicity in BAL cells, lung and liver tissue (tail length and % tail DNA), and relative *Saa1* (liver) or *Saa3* (lung) mRNA levels measured at 1-day post-exposure as extracted from Bendtsen et al. (2020). A cutoff of *r*_s_ ≥ 0.5 was used to identify correlations of interest.

#### Evaluation of differentially expressed proteins

Evaluation of differentially expressed proteins was determined based on dose–response relationships using linear models by the *lm* function in* R* for exposures with more than one dose group (all except CB). Vehicle control was considered as dose group 0. In the case of no entire dose-groups being above LOD in an exposure group, these proteins were not considered statistically significant despite *p* < 0.05 in linear model. In BALF, this was the case for four proteins in RME13 (IL17A, IL1B, IL-6 and PDGFB), four proteins in DEP13 (EDA2R, IL17A, IL1B and IL-6) and one protein in DEP17 (IL17A) (marked in Table S2). No additional proteins were excluded in plasma. Differentially expressed proteins following exposure to CB were evaluated by two-tailed *t* test versus vehicle control. In addition, the difference between the highest dose (54 µg) and vehicle control was evaluated by t test, and the results for this comparison are included in the dot plots.

#### Downstream pathway analysis on the differentially expressed proteins in BALF and plasma

Downstream pathway analysis was performed using the Ingenuity Pathway Analysis software (Qiagen, version 107,193,442). Input data were all differentially expressed proteins (*p* < 0.05) for each treatment in the linear models (RME13, HVO13, DEP13, DEP17) or two-tailed *t* test (CB) for BALF and plasma. In plasma, proteins were only considered for DEP13 and RME13 but not DEP17, HVO13 or CB due to insufficient number of significantly differentially expressed proteins (*n* ≤ 3). Fold change for highest dose (54 µg) for all treatments versus control was calculated and served as input for directionality of the protein changes and facilitated the prediction of the activation status of the denoted pathways/functions.

## Results

### Differentially expressed proteins in BALF and plasma

Differentially expressed proteins in BALF and plasma were determined based on dose–response relationships in a linear model for the combustion particles (RME13, HVO13, DEP13, DEP17) and by two-tailed *t* test for CB (Table 2). Full output from the linear models and *t* test can be found in Table S2 (BALF) and Table S3 (plasma). Exposure to HVO13 resulted in the greatest number of differentially expressed proteins (*p* < 0.05) in BALF (33), followed by DEP17 and DEP13 (24 and 22, respectively). The exposure to RME13 resulted in the lowest number of differentially expressed proteins in BALF (13). Following FDR adjustment for multiple comparison, the number of differentially expressed proteins were 31, 22, 21, and 8 for HVO13, DEP13, DEP17, and RME13, respectively. The CB particles induced seven differentially expressed proteins based on *t* test (and none after FDR adjustment) (Table 2).

**Table 2:** Number of differentially expressed proteins in BALF or plasma 24-h post-instillation exposure to renewable (RME13, HVO13) or petroleum (DEP13, DEP17) diesel exhaust particles

	Proteins in BALF (n)		Proteins in plasma (n)	
Exposure (doses)	*p* value < 0.05	FDR *p* value < 0.05	*p* value < 0.05	FDR *p* value < 0.05
RME13 (6, 18, 54 μg)	13	8	52	49
HVO13 (6, 18, 54 μg)	33	31	3	0
DEP13 (6, 18, 54 μg)	22	22	16	0
DEP17 (18, 54 μg)	24	21	3	0
CB (54 μg)	7	0	2	1

Carbon black (CB) particles were included as reference particles. Differentially expressed proteins were evaluated by linear models (RME13, HVO13, DEP13, DEP17) or *t* test (CB) and results shown as number of significant proteins based on *p* value (*p* < 0.05) and after adjustment for multiples testing (FDR-adjusted *p* value < 0.05)

RME13 = rapeseed methyl ester at 13% O_2_ engine intake; HVO13 = hydrogen-treated vegetable oil at 13% O_2_ engine intake; DEP13 = MK1 ultra-low-sulfur diesel at 13% O_2_ engine intake; DEP17 = MK1 ultra-low-sulfur diesel at 17% O_2_ engine intake; *CB* carbon black, *FDR* false discovery rate, *BALF* bronchoalveolar lavage fluid

**Fig. 1:**
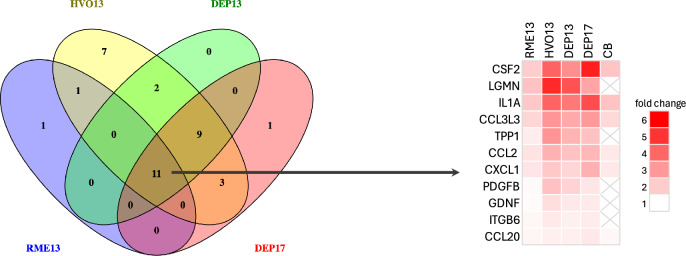
Venn diagram showing the overlap of differentially expressed proteins in BALF in response to exposure to renewable (RME13, HVO13) or petroleum (DEP13, DEP17) diesel exhaust particles on day 1 post-instillation in mice. Protein expression was measured by Proximity Extension Assay (Olink) and differentially expressed proteins were identified based on significant dose–response relationships (*p* < 0.05) in a linear model. Eleven proteins were differentially expressed by all diesel exhaust particles and are displayed in a heatmap based on fold change values of the highest dose (54 μg) compared to the vehicle control. Proteins are arranged in descending order based on the sum of the fold change for all treatments. For comparison purposes, differentially expressed proteins by carbon black (CB, 54 μg) based on a *t*-test (*p* < 0.05) are also included in the heatmap. With ‘X’ are marked the proteins that are not statistically significant

**Fig. 2:**
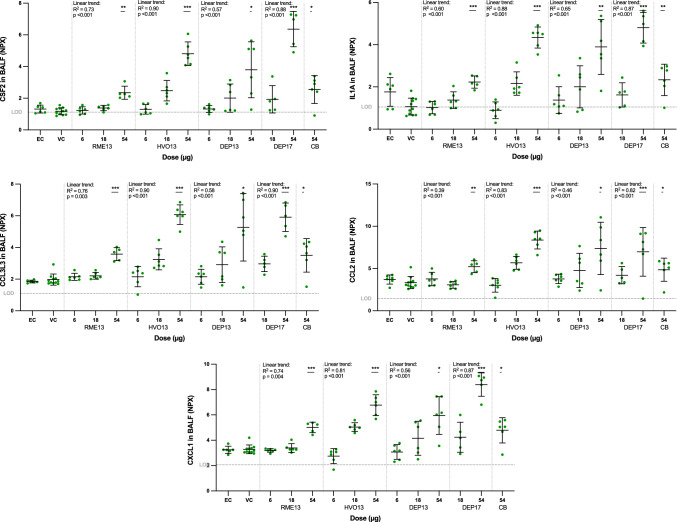
Protein expression in bronchoalveolar lung fluid (BALF) of mice 24 h post instillation exposure to renewable (RME13, HVO13) or petroleum (DEP13, DEP17) diesel exhaust particles. Carbon black (CB) particles were included as reference particles. Protein expression was measured by Proximity Extension Assay (Olink) and expressed as normalized protein expression (NPX) values on a log2-scale. *EC*, extraction control; *VC*, vehicle control

Eleven proteins were found to be differentially expressed (*p* < 0.05) and had a positive dose–response in BALF for all combustion particles (RME13, HVO13, DEP13, DEP17) investigated: CCL2, CCL20, CCL3L3, CSF2, CXCL1, GDNF, IL1A, ITGB6, LGMN, TPP1 and PDGFB (Fig. 1, Venn diagram). This group of proteins can, therefore, be considered as a signature protein fingerprint for (renewable) diesel-generated particles in BALF. RME13 had the lowest average fold change for the fingerprint proteins (calculated as ratio of the highest dose 54 µg versus vehicle control) of 1.6 while HVO13, DEP13, and DEP17 had relatively similar average fold changes of 2.8, 2.4, and 2.6, respectively (Fig. 1, heatmap). Six of the common proteins, *i.e.,* CSF2, IL1A, CCL3L3, CCL2, CXCL1, and CCL20, were also altered by CB with an average fold change of 1.7 (Fig. 1, heatmap). Dot plots of a selection from the fingerprint proteins are visualized in Fig. 2 (for proteins that were correlated with the in vivo toxicity endpoints percentage DNA in tail in BAL cells and neutrophil influx; see *Correlation between protein expression in BALF and plasma and *in vivo* endpoints*); the other proteins are visualized in Fig. S1.

Overall, fewer proteins were found to be differentially expressed in plasma compared to BALF for all tested particles except those derived from RME13, which resulted in 52 differentially expressed proteins (49 after FDR adjustment) (Table 2). Exposure to DEP13 induced 16 differentially expressed proteins in plasma, whereas HVO13 and DEP17 both resulted in 3 differentially expressed proteins. Exposure to CB particles resulted in two differentially expressed proteins in plasma. There were no common proteins in plasma between all combustion particles (Fig. S2) but seven proteins, *i.e.,* TGFB1, HGF, CLMP, PDGFB, TGFBR3, CXCL9 and DCTN2, were common between RME13 and DEP13 (dot plots in Fig. S3). In addition, one protein, CXCL1 in plasma was common for RME13, HVO13, and DEP17 (Fig. S3). In general, the estimate of the dose–response relationships (beta) was lower in plasma compared to BALF which was also reflected by the fold change for the highest dose that ranged between 1.1–1.4 (for the upregulated proteins).

### Pathway enrichment analysis

**Fig. 3:**
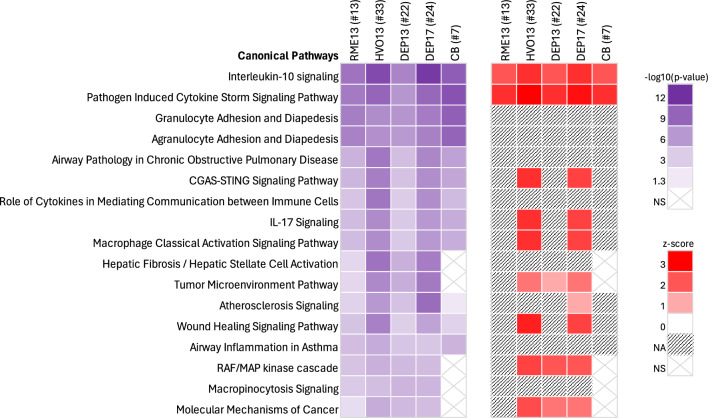
Top canonical pathways enriched related to protein changes in bronchoalveolar lung fluid (BALF) of mice 24 h post instillation exposure torenewable (RME13, HVO13) or petroleum (DEP13, DEP17) diesel exhaust particles. Carbon black (CB) particles were included as reference particles. Pathway analysis was performed in IPA on differentially expressed proteins based on dose–response relationships (linear model) or two-tailed t-test (CB). The total number of differentially expressed proteins that was input in the analysis is included in brackets after the respective exposure. Pathways are ordered according to the sum of statistical significance of the enrichment (expressed as –log10(*p*-value)). Some pathways are additionally characterized by z-score, a measure of the activation state of the pathway. A positive z-score is predictive of pathway activation. *NA* pathway activity score not available; *NS* pathway enrichment not significant for the respective exposure

Figure 3 shows the top ten enriched canonical pathways in BALF for each exposure combined into a heatmap. Overall, there was a large overlap in the top pathways for all exposures in BALF and many of the proteins were shared between the pathways. ‘Interleukin-10 signaling’ followed by ‘pathogen induced cytokine storm signaling’ were the two top enriched pathways for all exposures. Pathways with available z-scores, all displayed positive scores (range 1—3) which suggests a predicted activation of the respective pathways. It should be noted that the hepatic fibrosis pathway is defined by proteins in general related to fibrotic processes (*e.g.,* PDGFB, HGF, FAS, IL1A) and are not necessarily liver specific, as the name suggests.

**Fig. 4:**
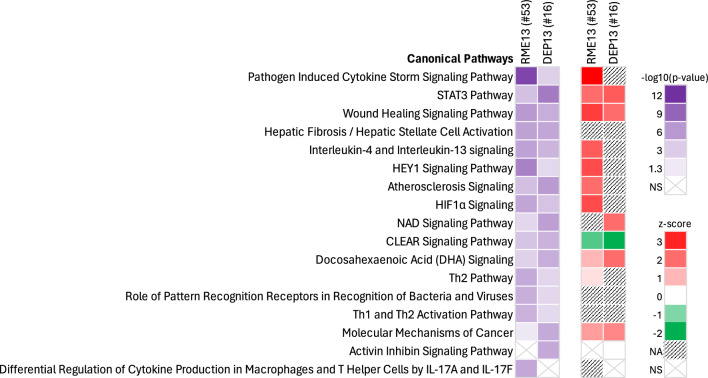
Top canonical pathways enriched related to protein changes in plasma of mice 24 h post instillation exposure to renewable (RME13) or petroleum (DEP13) diesel exhaust particles. Pathway analysis was performed in IPA on differentially expressed proteins based on dose–response (linear model). The total number of differentially expressed proteins is included in brackets after the respective exposure. Pathways are ordered according to the statistical significance of the enrichment (expressed as –log10(*p*-value)). Some pathways are additionally characterized by z-score, a measure of the activation state of the pathway. A positive z-score is predictive of pathway activation and a negative z-score is predictive of pathway inhibition. *NA* pathway activity score not available; *NS* pathway enrichment not significant for the respective exposure

The number of proteins differentially expressed for HVO13 and DEP17 in plasma was too low for downstream analysis, and we, therefore, proceeded with pathway and network analysis only for RME13 and DEP13. Similar to the response in BALF, enriched pathways were primarily related to cellular immune system as well as cellular stress and injury (Fig. 4). Some of the top pathways were found to be common between BALF and plasma, namely ‘pathogen induced cytokine storm signaling pathway’, ‘wound healing signaling pathway’, ‘hepatic fibrosis/hepatic stellate activation’, and ‘tumor microenvironment pathway’.

**Fig. 5:**
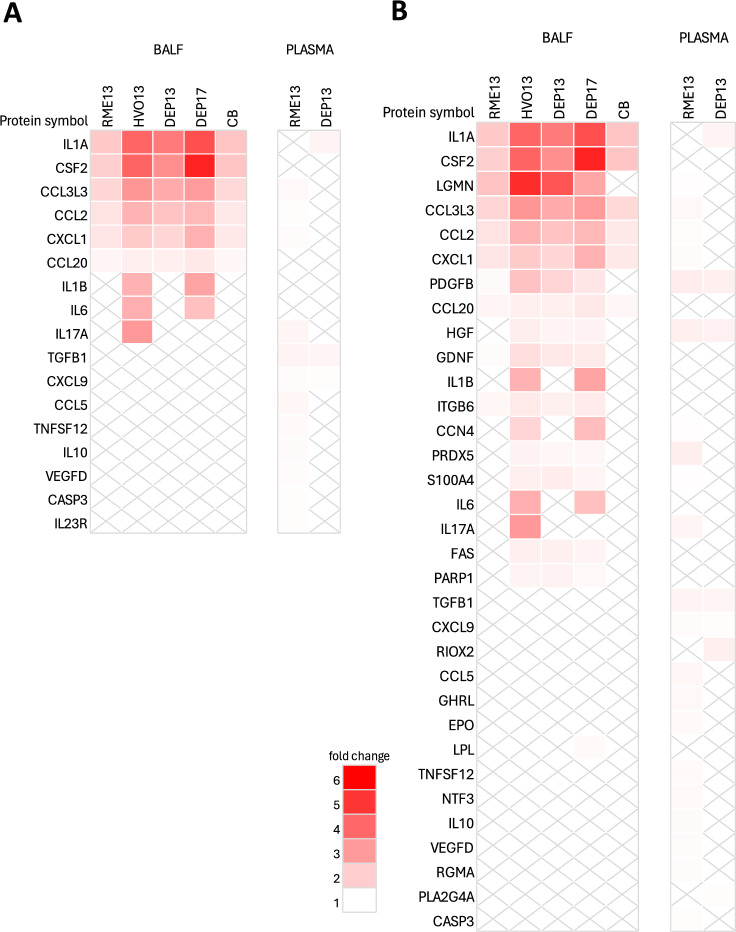
Protein changes for proteins included in the canonical pathway” pathogen induced cytokine storm signaling pathway” (**A**) or the function”inflammatory response” (**B**) in bronchoalveolar lung fluid (BALF) or plasma of mice 24 h post instillation exposure to renewable (RME13, HVO13) or petroleum (DEP13, DEP17) diesel exhaust particles. Carbon black (CB) particles were included as reference particles. Results for plasma following exposure to HVO13, DEP17 and CB were excluded from the analysis due to very low number of differentially expressed proteins. Fold change refer to fold change of the highest dose (54 μg) compared to the vehicle control. Proteins are arranged in descending order based on the sum of the fold change for all treatments. With’X’ are marked the proteins that are not statistically significant

The proteins included in the canonical pathway ‘pathogen induced cytokine storm signaling pathway’ are visualized in Fig. 5A in terms of fold change of the highest dose (54 µg) versus vehicle control. The pathway is denoted by proteins such as CCL2, CXCL1, and IL1A and was enriched and predicted activated in response to all exposures in BALF as well as to RME13 in plasma. However, the specific proteins and the size of the effect differed between the matrices. In BALF, the average fold change for proteins within this pathway was relatively similar for HVO13 (2.8), DEP13 (2.5), and DEP17 (3.1). RME13 and CB had a lower protein fold change, on average 1.7. RME13 and DEP13 also showed an enrichment of the ‘pathogen induced cytokine storm signaling pathway’ in plasma yet the protein profiles within the pathway differed. Three proteins within this pathway were found to be common in both BALF and plasma in response to RME13 (CCL2, CXCL1, and CCL3L3), but otherwise the different matrices displayed changes in different proteins. The size of the change was also lower in plasma compared to BALF with an average fold change of 1.1.

Among the top functional networks, ‘inflammatory response’ and ‘chemotaxis’ were enriched and predicted to be activated by all exposures in BALF, and the pattern of the fold changes for the proteins was similar with the ones observed in the pathway analysis. Figure 5B shows a heatmap of the corresponding proteins for the ‘inflammatory response’ network in both BALF and plasma. Figure S4 shows overlapping networks between ‘inflammatory response’ and ‘chemotaxis’ for the exposures and different matrices.

### Correlation between protein expression in BALF and plasma and in vivo endpoints

**Fig. 6:**
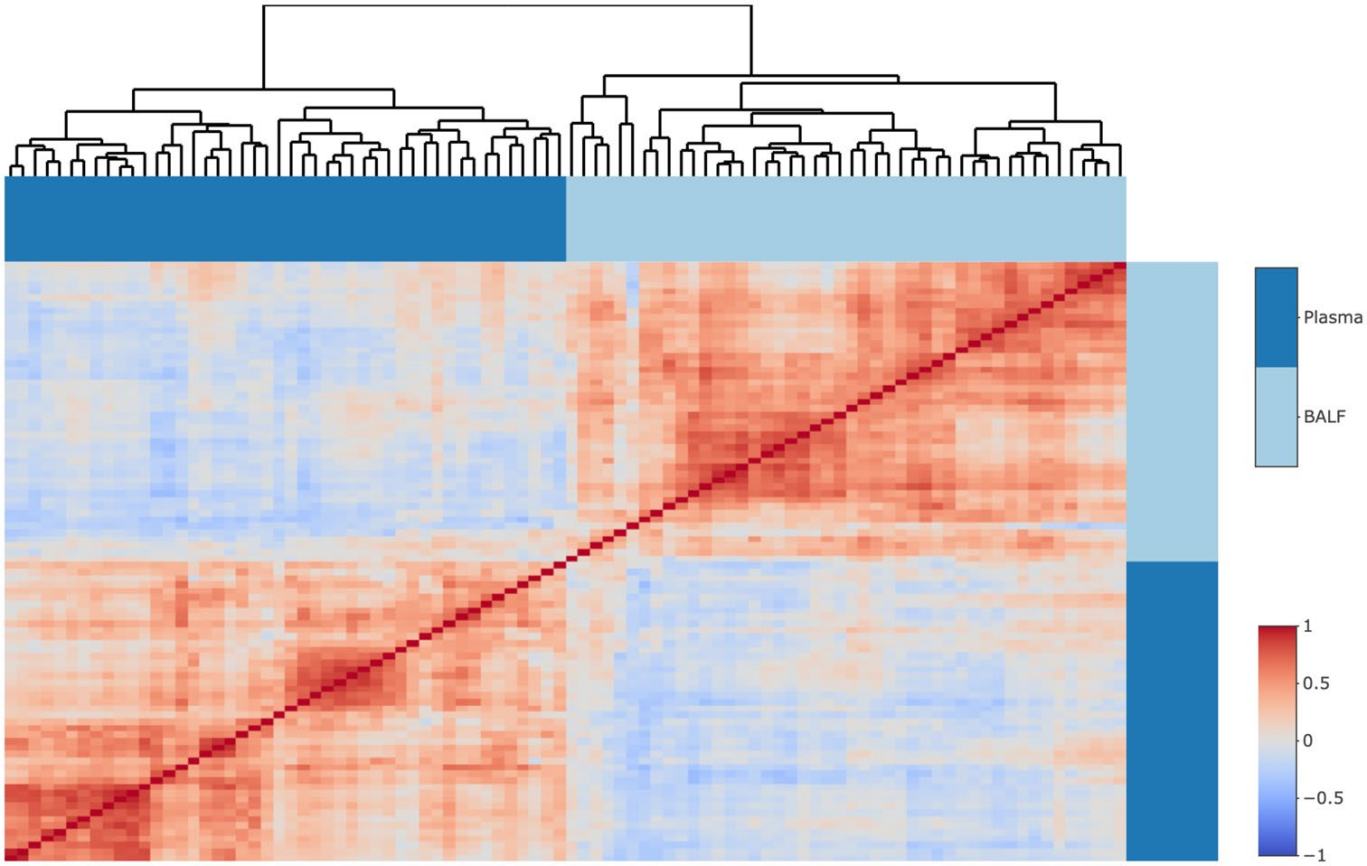
Correlation (Spearman) heatmap between protein expression in plasma and bronchoalveolar lung fluid (BALF) in mice 24 h post instillation exposure to renewable (RME13, HVO13) or petroleum (DEP13, DEP17) diesel exhaust particles. Carbon black (CB) particles were included as reference particles. Protein expression was measured by Proximity Extension Assay (Olink) and expressed as normalized protein expression (NPX) values on a log2-scale

A heatmap of the correlation (Spearman) between proteins in BALF and plasma is displayed in Fig. 6. Proteins in plasma and BALF formed two separate clusters. Overall, high intercorrelations were found within BALF as well as within plasma. None of the proteins had a correlation between plasma and BALF that was higher than 0.5. However, CXCL1 in BALF and plasma was found to be significantly (*p* < 0.05) correlated (*r*_S_ = 0.35), see Fig. S5.

**Fig. 7:**
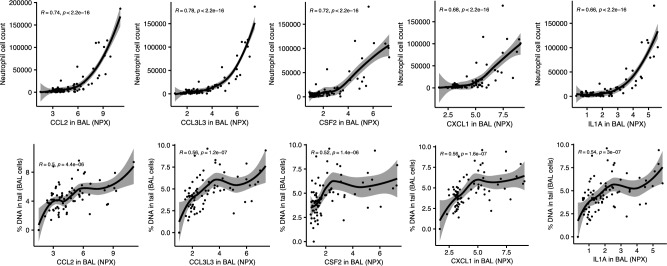
Correlation (spearman) plots between protein expression in bronchoalveolar lung fluid (BALF) and in vivo biomarkers of inflammation (neutrophil cell counts) and genotoxicity (%DNA in tail) in mice 24 h post instillation exposure to renewable (RME13, HVO13) or petroleum (DEP13, DEP17) diesel exhaust particles. Carbon black (CB) particles were included as reference particles. Protein expression was measured by Proximity Extension Assay (Olink) and expressed as normalized protein expression (NPX) values on a log2-scale. Line represents the loess curve and shading stands for the confidence interval

Moderate correlations (*r*_S_ ≥ 0.5) were identified between proteins detected in BALF and two in vivo toxicity endpoints measured at 24-h post-exposure, *i.e.,* DNA damage measured as percentage DNA in tail in BAL cells and neutrophil influx, a biomarker of inflammation. Five proteins in BALF correlated with percentage DNA in tail in BAL cells, namely CCL2, CCL3L3, CSF2, CXCL1, and IL1A (Fig. 7). Thirteen proteins in BALF correlated with neutrophil influx: CCL2, CCL20, CCL3L3, CSF2, CXCL1, GDNF, IL17A, IL1A, IL1B, IL-6, LGMN, PDGFB, and TPP1 (Fig. 7 shows the five proteins that overlapped with the proteins correlated with percentage DNA in tail in BAL cells, and Fig. S6 shows the rest of the proteins). No correlations *r*_S_ ≥ 0.5 were identified between proteins in plasma and in vivo toxicity endpoints. The highest correlations for proteins in plasma were for PLXNA4 (*r*_S_ = 0.41) and CXCL9 (*r*_S_ = 0.34) and comet tail length in liver tissue. (Fig. S7).

## Discussion

### Comparative toxicity of the (bio)diesel exhaust particles and association with physicochemical properties

The currently available in vitro and in vivo literature on the comparative toxicity between renewable and petroleum diesel exhaust is inconclusive (Godri Pollitt et al. 2019; Møller et al. 2020), as increased, comparable, and decreased potency has been reported. A qualitative relative comparison of the physicochemical properties and in vivo responses after exposure to renewable or petroleum DEPs is displayed in Table 3. The results of our study suggest that exhaust particles derived from HVO13 induce the highest number of altered proteins in BALF, followed by DEP17 and DEP13, whereas RME13 and CB particles exhibit least effects. When it comes to the size of the effect, HVO and DEPs were relatively similar in potency, while RME13 and CB induced relatively small (although significant) changes. This is in line with the data from Bendtsen et al. (2020) where a similar pattern was observed for neutrophil influx as a marker for lung inflammation.

**Table 3:** Qualitative relative comparison of physicochemical and toxicity data from combustion of renewable (RME13, HVO13) or petroleum (DEP13, DEP17) diesel exhaust particles

Physicochemical properties	RME13	HVO13	DEP13	DEP17	CB
Oxygen content in fuel (%)	10	0	0	0	–
PAHs	−/ +	+ +	+	−/ +	–
OC	+ +	+ +	+	+ +	–
EC	+ +	+ +	+ + +	+	+ + + +
ROS	+ +	+ + +	+ +	+ + +	+ + +
Metals	+ +	+ +	+	+ + +	–
Surface Area	+ + +	+ +	+ +	+ +	+ + + +
In vivo response (Day 1)					
Neutrophils and lymphocytes in BAL	−/ +	+	+ +	+	−/ +
Acute-phase response (lung *Saa3* mRNA)	−/ +	+ + +	+ + +	+ +	+
DNA damage (% DNA tail in BAL cells)	–	–	–	–	–
Protein response in BALF as number of proteins	+	+ + +	+ +	+ +	+
Protein response in BALF as effect size	+ +	+ + +	+ + +	+ + +	+ +
Protein response in plasma as number of proteins	+ + +	–	+	–	–
Protein response in plasma as effect size	+	–	+	–	–

Table modified from Bendtsen et al. (2020) with the addition of the protein data generated in this study. RME13 = rapeseed methyl ester at 13% O_2_ engine intake; HVO13 = hydrogen-treated vegetable oil at 13% O_2_ engine intake; DEP13 = MK1 ultra-low-sulfur diesel at 13% O_2_ engine intake; DEP17 = MK1 ultra-low-sulfur diesel at 17% O_2_ engine intake; BALF = bronchoalveolar lavage fluid; PAH = polycyclic aromatic hydrocarbons; OC = organic compounds; EC = elemental carbon; ROS = reactive oxygen species; relative categories: “–” = no content/response, “-/ + ” = very low content/response, “ + ” = low content/response, “ +  + ” = medium content/response, “ +  +  + ” = high content/response

It is well-established that the toxic potency of particles is related to their physicochemical properties. While the size and estimated specific surface area were relatively comparable between the tested particles, the content and composition of PAHs differed. The total content of PAHs was the highest for particles derived from HVO13, lower for DEP13, and the lowest for RME13 and DEP17. Since HVO13 showed the largest number of differentially expressed proteins, it is plausible that PAH content might contribute to some of the differences in protein changes in BALF. This could be mediated by PAH binding and activation of aryl hydrocarbon receptor (AHR) that may result in induction of enzymes such as CYP1A1 and ultimately increase generation of reactive oxygen species and inflammation (Vogel et al. 2020). In addition, to further support that hypothesis, the CB particle that were used as reference are free from organic components and induced the lowest number of differentially expressed proteins in BALF. No significant DNA damage in BAL cells was observed at day 1; however, such effects were observed at later timepoints (Bendtsen et al 2020). While several PAHs are classified as class 1 human carcinogens by IARC (IARC 2010), the PAH fraction from air pollution has also been proposed to contribute to non-malignant respiratory diseases via mediation of processes such as inflammation and epithelial/endothelial function (Låg et al. 2020).

All tested particles had a relatively high ROS generation capacity, with the highest potential being for CB, DEP17, and HVO13, and a relatively lower potential for RME13 and DEP13 (by mass). Unfortunately, in this study, we did not test particles with a low generation capacity (for example, the DEP9.7 particles used in Bendtsen et al. (2020)), and it is, therefore, difficult to draw any conclusions on the ROS contribution to the observed effects. Moreover, the CB particles are known to be potent ROS generators (Di Ianni et al. 2022) but induced relatively few and low protein alterations in BALF. While ROS capacity could not be linked to the protein changes in BALF at day 1, particle-generated ROS correlated with particle-induce genotoxicity at 28- and 90-day post-exposure as shown in Bendtsen et al (2020).

RME contains a substantial amount of oxygen (10%) in the fuel in the form of methyl esters. Aerosol mass spectrometry data from a later campaign at 17% oxygen intake, but at similar loads, show that the organic aerosol emissions from RME have a higher O:C ratio and a lower H:C ratio compared to DEP (Novakovic et al. 2024) indicating presence of fuel-related degradation products. We hypothesize that fuel influences the organic aerosol composition and potentially also soot surfaces, which might affect the kinetics of the particles in the lung.

One of the main benefits of renewable diesel fuels is the reduction in the amount of generated particulate matter. However, in this study, exposure was done at the same mass concentrations to facilitate hazard comparison between the particles. The renewable fuels have an approximately three times reduction in the emitted particles mass which would be equivalent to comparing the dose of 18 μg for HVO13 and RME13 to 54 μg for DEP13. Therefore, while the overall hazard was found to be similar between the particles, renewable fuels have potential to reduce exhaust emissions, and thereby exposure which is likely to lessen associated health risks.

### Protein fingerprint for combustion particles in BALF and mechanisms of lung toxicity

The 92 proteins within the analyzed panel are linked to pathways related to cellular inflammation, but not exclusively; a large portion of the proteins were also related to cell movement, cardiovascular disease, and cancer. By quantifying this protein panel in BALF, we could confirm the inflammatory effects observed in the lungs of the same animals, as reported in Bendtsen et al. (2020), but also achieve a higher mechanistic resolution.

We identified 11 key proteins that were associated with exposure to the (bio)diesel combustion particles in BALF in a dose–response matter, independent of fuel type used. These proteins can be considered as a signature fingerprint for acute (bio)diesel combustion particles exposure in BALF. The majority fingerprint proteins are inflammatory cytokines and/or chemokines (CCL2, CXCL1, CCL3L3, CSF2, IL1A, CCL20). The CC chemokines primarily participate in the migration and activation of monocytes, macrophages, and lymphocytes, whereas those belonging to the CXC family specifically attract neutrophils (Baggiolini, 2001). IL1A is a cytokine which regulates multiple pathways of the inflammatory cascade and secondary inflammatory mediators such as chemokines that in turn regulate cell recruitment to the inflammatory site. IL1A has previously been associated with exposure to DEP in vitro (Grilli et al. 2018) and has been shown to mediate acute lung injury in mice after exposure to other types of particles such as silica (Rabolli et al. 2014). CXCL1 is a chemoattractant chemokine that plays a major role in mediating acute inflammatory response. CXCL1 has a high level of homology with CXCL2 (80%), and they activate the same receptor CXCR2. CXCL2 has been linked to the inflammatory process in response to particles (Driscoll, 2000) and has previously been suggested as a key player for early DEP-induced neutrophilic inflammation (Saber et al. 2006). We showed that IL1A and other chemokines such as CXCL1 were associated with neutrophil cell count in BALF, a marker of lung inflammation. This is in line with chemokines being neutrophil chemoattractants. To note, all particles except for RME13 induced pulmonary inflammation in terms of increased neutrophil cell count (Bendtsen et al 2020), which indicates once again that protein changes in BALF could be sensitive markers of lung inflammation and they occur prior to the neutrophil influx. This is to be expected since the chemokines are involved in recruiting immune cells to the site of injury. Additional proteins included in the fingerprint included LGMN, which is involved in processing of proteins for antigen presentation in the lysosomal/endosomal system and is associated with tumor development and invasion (Khan et al. 2023). Another protein was ITGB6, a receptor for fibronectin among others and is involved in wound healing and fibrosis (Meecham and Marshall 2020). *Lgmn* and *Itgb6* gene expression in the lung was shown to be upregulated after exposure to multi-walled carbon nanotubes in mice (Poulsen et al. 2015) but no literature was found in relation to other combustion particles. Gene expression of *Itgb6* in lung tissue of mice was previously reported to be upregulated after instillation exposure to carbon black (5 days post-exposure 162 µg) (Husain et al. 2015). TPP1 is a multi-purpose peptidase (Tomkinson, 2019) but with no reports of association with particle exposure. GDNF is a growth factor important for neuronal development, and expression in the lung is increased in asthma rat models (Wang et al. 2023). No report of GDNF associations with exposure to particulate matter was found. PDGFB is involved in airway remodeling (Kardas et al. 2020) and increased secretion of PDGFB was observed in vitro macrophages after exposure to diesel exhaust particles and it was suggested to be mediated by activation of AhR (Jaguin et al. 2015). Interestingly, PAH-mediated activation of AhR receptor has been suggested to play a role in vascular toxicity from exposure to PM 2.5 (Ho et al. 2022).

Most of the identified fingerprint proteins were also highly correlated with in vivo inflammatory marker of neutrophil cell count (all except ITGB6). This protein signature fingerprint may not be unique to particles of diesel exhaust yet represents the local pathophysiological response induced as a result of exposure. The relevance of the fingerprint in BALF for other types of combustion particles remains to be investigated. The six inflammatory cytokines/chemokines found in the fingerprint were also altered by exposure to reference carbon black particles. This suggests that insoluble nano-sized carbon particles reflecting the carbon core of diesel exhaust particles contribute to the local lung inflammatory effects. CB particles were shown to induce lung inflammation in vivo in other studies (Bourdon et al. 2012).

One of the top enriched pathways in BALF for all exposures was the ‘pathogen induced cytokine signaling pathway’. This pathway includes components of the acute phase response such as IL1A, IL1B and IL-6 and encompassed multiple genes present in other enriched pathways such as ‘interleukin 10 signaling’, ‘airway pathology in COPD’, ‘roles of cytokines in mediating communication between immune cells’, cell movement related pathways ‘granulocyte/agranulocyte adhesion and diapedesis’. Apart from the immune/inflammation-related pathways, the analysis indicates enrichment of pathways related to fibrosis and extracellular matrix signaling, *i.e.,* ‘(hepatic) fibrosis’, ‘wound healing signaling pathway’ and ‘tumor microenvironment’. Specific proteins from these latter pathways are TGFα (upregulated by HVO), ITGB6, and PDGFB (upregulated by all combustion particles and part of the protein fingerprint). Development and progression of lung fibrosis is a well-established response to particle exposure and it is mediated by a complex interplay between inflammation, oxidative stress, fibroblast activation, and epithelial–mesenchymal transition (Bonner, 2007). Recently, OECD approved an adverse outcome pathway for fibrosis, where chronic inflammation is one of the key events, highlighting the role of inflammation in fibrosis (Halappanavar et al. 2023).

### Protein changes in plasma and systemic effects

The occurrence and potential role of acute systemic inflammation following pulmonary exposure to particulates including DEPs is inconsistent in the literature. The results of our study demonstrated a rapid release of inflammatory mediators into the systemic circulation following pulmonary exposure to RME13 exhaust particles, and to some extent to DEP13, but not to the other tested particles. It is, however, unclear whether the other DEP exposures would increase systemic response at a later time point, in that they could exhibit a delayed response. Regarding the effect size of the changes, these were on average substantially lower in plasma compared to BALF. This is to be expected since the particles responsible for the observed effects are to a large extent still in the lung after 24 h. It is unclear how these changes develop over time, together with particle clearance and (to a lesser extent) particle translocation. Exhaust particles derived from RME were shown to have less particle retention in the lung by day 28, which could possibly indicate a more rapid clearance for DEPs from RME fuel compared to HVO and petroleum diesel (Bendtsen et al. 2020). It is also unclear to which extent such relatively small changes in plasma are biologically relevant. It is plausible that small changes in critical proteins that occur over prolonged periods of time (repeated exposures in for example occupational or traffic-dominated polluted environments) could result in prolonged low grade effects that will ultimately lead to adverse outcomes.

Interestingly, the enrichment pattern of pathways in plasma for RME13 was in general similar to what was observed in BALF but the number of proteins was higher. Notably, RME13 activated the ‘HIF1α pathway’ in plasma which could have implications for cardiovascular outcomes via cardiac remodeling (Sato and Takeda 2023).

Acute-phase response is a non-specific early response to infection and inflammation (Gabay and Kushner 1999). Increasing evidence suggests that pulmonary exposure to particles triggers an acute phase response, which may be part of the causal link between particle exposure and risk of cardiovascular disease (Hadrup et al. 2020). The alterations of neutrophil numbers, increased *Saa3* mRNA in lung and SAA3 plasma protein abundance have been correlated in mice in response to particle exposure and suggested as interchangeable biomarkers of particle-induced acute phase response in animal models (Gutierrez et al. 2023). Upregulated *Saa3* expression in lung of mice exposed to exhaust particles derived from HVO13, DEP13, and DEP17, but not RME13, was previously reported 24-h post-exposure (Bendtsen et al. 2020). Unfortunately, neither of the acute-phase response proteins SAA and CRP were analyzed in this study in plasma due to limited availability of biological material. Yet, acute-phase proteins are released as a result of inflammatory cytokines such as interleukin 1β and 6 (IL-1β, IL-6), which are main upstream mediators of acute phase response and regulate the secretion of acute-phase proteins (Gabay and Kushner 1999). In this study, protein expression of IL-1β and IL-6 was found to be upregulated in BALF in response to HVO13 and DEP17, but not RME13 or DEP13. However, low correlation (*r*_s_ < 0.5, p < 0.05) was found between protein abundance in BALF and *Saa3* expression in lung tissue. Yet, IL-1β and IL-6 in BALF were found to be correlated with neutrophil count, which in turn was correlated to *Saa3* in lung, as described in Bendtsen et al. (2020). Also, it was previously shown that pulmonary exposure to diesel particle NIST2975 increased plasma levels of SAA3 (Bendtsen et al. 2019).

In a review by Saber et al. (2014), it is concluded that pulmonary exposure to particles results in a large and robust pulmonary acute-phase response to a larger degree compared to a hepatic acute-phase response. In line with this, no effects were observed in the expression of acute-phase regulator proteins IL-1β and IL-6 in plasma in response to any exposure in this study. Repeated inhalation exposure (4 days) to DEPs in mice has previously been shown to not induce systemic acute-phase response (*Sap* or *Saa* in liver or SAA plasma concentration) at exposure concentrations inducing substantial pulmonary inflammation (Saber et al. 2009). In a controlled inhalation study where humans were exposed to HVO diesel exhaust for 3 h, no alterations of inflammatory proteins (*n* = 92) were observed in plasma (Krais et al. 2021). Important to note is though that the study only investigated short-term exposure with low exhaust concentrations being exposed to healthy human subjects. Systemic inflammatory effects in the form of increased plasma concentrations of IL-6 and TNFα as a result of DEPs (SRM 2975) have been reported in Wistar rats by 24-h post intratracheal instillation exposure (0.5 mg) (Robertson et al. 2012).

While we did not observe a common fingerprint in plasma, there were seven proteins (TGFB1, HGF, CLMP, PDGFB, TGFBR3, CXCL9 and DCTN2) that were upregulated by both exposure to RME13 and DEP13. Out of these, PDGFB was also part of the protein fingerprint in BALF as discussed in the protein fingerprint in BALF section. Also, HGF was upregulated by HVO13, DEP13, and DEP17 in BALF. HGF or the hepatocyte growth factor is an important mediator of acute and chronic inflammation and is involved in suppression of inflammatory immune responses (Molnarfi et al. 2015). HGF is involved in lung tissue repair (Panganiban and Day 2011) and air pollution (NO_2_ levels in particular) were found to be associated with an increase in blood levels of HGF in COPD patients (Dadvand et al. 2014). In addition, pulmonary exposure to ultrafine carbon black particles in mice leads to an increase in HGF levels in BALF and subsequent proliferation of type II alveolar epithelial cells. The other proteins that were specific to plasma are briefly discussed below.

CLMP is a protein part of the epithelial tight junction and involved in cell–cell adhesion (Raschperger et al. 2004). CLMP is involved in inflammatory response of cardiac fibroblasts during myocardial infarction (Han et al. 2020). Data on effects on CLMP following exposure to particles in general were scarce but we found one study that showed increased gene expression of CLMP following acute exposure of BEAS-2B cells to diesel combustion particles (Grilli et al. 2018). TGFB1 is a pro-fibrotic growth factor and a master regulator of lung fibrosis (Aschner and Downey 2016). Interestingly no effects were observed in BALF, only increase in expression in plasma for RME13 and DEP13. Exposure of mice to PM2.5 particles resulted in increased Tgfb1 gene expression in lung tissue (Zeng et al. 2024). In addition, a dose-dependent increase in gene expression of Tgfb1 was observed both in the lung and PBMCs of rats exposed to DEP (NIST 2975) via intratracheal instillation (Srivastava et al. 2014). TGFBR3 is co-receptor for TGFB molecules and is involved in TFGB signaling; but other more independent roles are emerging (Vander Ark et al. 2018). No association between TGFBR3 and diesel combustion particle exposure could be identified. CXCL9 is a cytokine involved in regulation of migration, activation, and differentiation of immune cells (Tokunaga et al. 2018). *Cxcl9* gene expression was downregulated in tissue residing alveolar macrophages after 4-week inhalation exposure to PM2.5 in mice (Gangwar et al. 2020). Similarly CXCL9 gene expression was downregulated in human PBMCs after 48-h exposure to coarse PM10 (Marín-Palma et al. 2023). Dynactin (DCTN) proteins are involved in intracellular transport, and high levels of DCTN2 are associated with a poor prognosis in hepatocellular carcinoma (Li et al. 2022). To our knowledge, no studies have associated DCTN2 expression with exposure to particulate matter. While many of the aforementioned proteins are coupled with progression of different diseases, none of the proteins in plasma acts as a well-established biomarker of risk or disease progression.

### Correlations between proteins in plasma and BALF as well as prediction of in vivo toxic endpoints

In this study, we show that single-dose instillation exposure to petroleum and renewable DEPs in mice was associated with short-term alterations in protein profiles of BALF to a greater extent than in plasma. This was expected since the particles are localized mostly in the lung and have a relatively low translocation after 24 h. The expression of several proteins in BALF showed a good correlation with in vivo inflammatory and genotoxic outcomes namely neutrophil cell count and DNA strand breaks in BAL cells. The observed correlation between cytokine levels and DNA strand break levels in BALF could suggest that the DNA damage is secondary genotoxicity caused by inflammation and oxidative stress or suggest that both are caused by a common factor such as particle-induced ROS.

Overall, assessing alterations of protein profile in BALF was shown to be a more sensitive method in predicting lung inflammation in response to DEPs, as compared to more conventional methods of bronchoalveolar lavage cell count. For example, the exposure to RME13 was not reported to induce an inflammatory or acute-phase response as presented in Bendtsen et al. (2020); however, increase in pro-inflammatory cytokines (*e.g.,* IL1A, CCL2, CXCL1) was detected in BALF in response to RME13 (and CB) albeit at a general lower magnitude compared to that exhibited by the other tested DEPs. The latter is in line with the previously reported prominent inflammatory response observed as increased neutrophil influx for HVO13, DEP13, and DEP17 (Bendtsen et al. 2020). In contrast, we found that proteins in plasma were poorly associated with markers of in vivo lung inflammation. The local inflammatory lung response did not result in corresponding acute alterations in the systemic circulation for petroleum or HVO-generated DEPs at the time point measured (24 h). Yet, we detected an increase in plasma inflammatory proteins after exposure to RME13 (and DEP13), which suggests a corresponding acute response by systemic tissues, which, if chronic and persistent, may trigger cardiovascular dysfunction.

Considering all exposures, one protein was found to be significantly correlated between BALF and plasma, namely CXCL1, albeit with a relatively low coefficient (*r*_s_ = 0.35). CXCL1 is a chemokine that mediates acute inflammatory response and is included in the signature fingerprint for DEPs in BALF as well as differentially expressed in plasma after exposure to exhaust particles derived from RME13, HVO13, and DEP17. CXCL1 could, therefore, potentially serve as a non-invasive biomarker for assessing the presence and severity of particle-induced local lung inflammation. However, the coefficient of correlation is relatively low, and further research is needed to validate its utility as a biomarker and to determine its sensitivity and specificity for different types of particle exposures.

### Strengths and limitations

A strength of this study was that we used biological material from the same experiment as Bendtsen et al (2020) which allows for the direct correlation of the different in vivo toxicity endpoints with the exploratory panel of proteins. In addition, the combustion particles were thoroughly characterized which enables the correlation of the toxicity with physicochemical characteristics. The experimental setup that allowed for the identification of dose–response relationships for the protein changes gives an additional weight to the findings.

A limitation of the study is the lack of protein measurements at later time points, which would have allowed a better understanding of the dynamic of the effects. It is, at present, unclear whether the observed changes in protein patterns are persistent and if the protein levels of the signature fingerprint remain altered after prolonged time post-exposure or if it solely reflects acute alterations in BALF. Also, it is not clear whether the effects observed in plasma at 24 h for RME13 would also be present for the other particles at later timepoints. The inflammatory effects in terms of neutrophil influx and *Saa3* expression in response to HVO13 and DEP17 were found to be largely resolved by day 28 or 90 post-exposure. However, DEP13 appeared to be more persistent inflammogenic than other DEP particles as *Saa3* expression was increased at 28 days and increased neutrophil and lymphocyte influx was still evident after 90 days (Bendtsen et al. 2020).

In our work, we used a targeted approach for quantification of proteins but we are aware that untargeted approaches can give additional information on proteins that were not included in our panel. Using untargeted proteomic approaches, Lewis et al. identified increase levels of inflammation-related proteins (e.g., anaphylatoxin C3a and calgranulin A) 24 h after pulmonary exposure to diesel exhaust particles (NIST1650) (Lewis et al. 2007). In the case of traditional proteomic approaches, the high-abundance proteins can mask the low-abundance proteins but this is less of a problem with the proximity extension assay used in the current project. Also, in this study, we worked with whole BALF samples without isolation of the extravesicular (EV) fraction. However, EV approaches can be useful to identify and quantify proteins with low abundance such as proteins related to cytoskeletal remodeling and lipid metabolism and is a promising approach for diagnostic and monitoring of lung disease such as idiopathic pulmonary fibrosis (Shaba et al. 2021). Other approaches have included evaluation of BALF exosome (subpopulation of extracellular vesicles) and found, for example, protein signatures consistent with lung inflammation after exposure to ozone in mice (Choudhary et al. 2021).

The measurements of proteins levels in plasma and BALF have not been validated by traditional methods such as immunoblotting, due to sample availability. However, the proximity extension assay is a well-established and highly sensitive immunoassay, previously used to identify biomarkers of progression cystic fibrosis in BALF of children (Horati et al. 2024) and biomarkers of disease in adults with interstitial lung disease (Majewski et al. 2021). A strength of our study is that protein data in BALF for several proteins (CCL2, CCL20, CCL3L3, CSF2, CXCL1, GDNF, IL17A, IL1A, IL1B, IL-6, LGMN, PDGFB and TPP1) had a good correlation (*r*_S_ > 0.5) with classical lung inflammation markers such as neutrophil influx.

When analyzing a large number of endpoints like in this study, issues such as multiple comparison testing become relevant due to the risk of chance findings. To account for that, we also present results after FDR correction, which is standard approach for adjusting *p* values. To note, however, the multiple comparison problem is relevant when the different comparisons are independent of each other. As our data show, proteins were highly correlated in BALF and plasma, respectively. Therefore, the independence of the multiple comparisons can be disputed, and there is a possibility that using the FDR method, we are over-adjusting and increasing the risk of false negative results. For those reasons, while we present both adjusted and non-adjusted results, the discussion and interpretation of our data are based on the non-adjusted *p* values.

Finally, when interpreting the pathway enrichment data, it is important to acknowledge that this is not a whole proteome study and we only analyzed 92 proteins which limits that pathways and functions that can be enriched.

## Conclusion

The results of this study show that single instillation exposure to exhaust particle generated from both renewable sources and petroleum induced acute upregulation of a common fingerprint of pro-inflammatory proteins in BALF in mice. Despite overlapping effects, particles generated from RME13 were less potent at inducing local inflammatory changes, but more potent in inducing systemic effects compared to HVO13 or conventional DEP13. The possible reasons behind this pattern for RME could be related to fuel properties and its higher oxygen content, which has been shown to alter soot formation and oxidation processes as well as composition of organic aerosol (Malmborg, 2020). Overall, we found that particles resulting from the combustion of renewable fuels may carry a comparable intrinsic toxicological hazard compared to those from conventional fuels. However, RME and HVO reduced particle mass emissions (per energy or fuel volume unit) by about a factor of 3 and consequently reduce exposure to particulate matter which should be considered in the context of health risk assessment.

Finally, the quantitative protein-based approach employed here proved to be a sensitive method for identifying distinct protein changes in different matrices as well as a promising method for identification of biomarkers of toxicity. This is highly relevant for gaining mechanistic understanding on particle toxicity in general and can reveal differences in toxicity pathways of common air pollutants if applied to a broad spectrum of emission sources.

## Supplementary Information

Below is the link to the electronic supplementary material.

Supplementary file1 (PDF 1296 KB)

Supplementary file2 (XLSX 17 KB)

Supplementary file3 (XLSX 26 KB)

Supplementary file4 (XLSX 34 KB)

## Data Availability

The datasets generated during and/or analysed during the current study are available from the corresponding author on reasonable request.
