# Supplementary material for: Pulmonary exposure to renewable diesel exhaust particles alters protein expression and toxicity profiles in bronchoalveolar lavage fluid and plasma of mice
Source: Arch Toxicol. 2024 Dec 29;99(2):797–814. doi: 10.1007/s00204-024-03915-y (PMC11775017; doi:10.1007/s00204-024-03915-y)
Supplement: Supplementary file 1 — Supplementary file1 (PDF 1296 KB) [file 204_2024_3915_MOESM1_ESM.pdf]

Supplementary figures

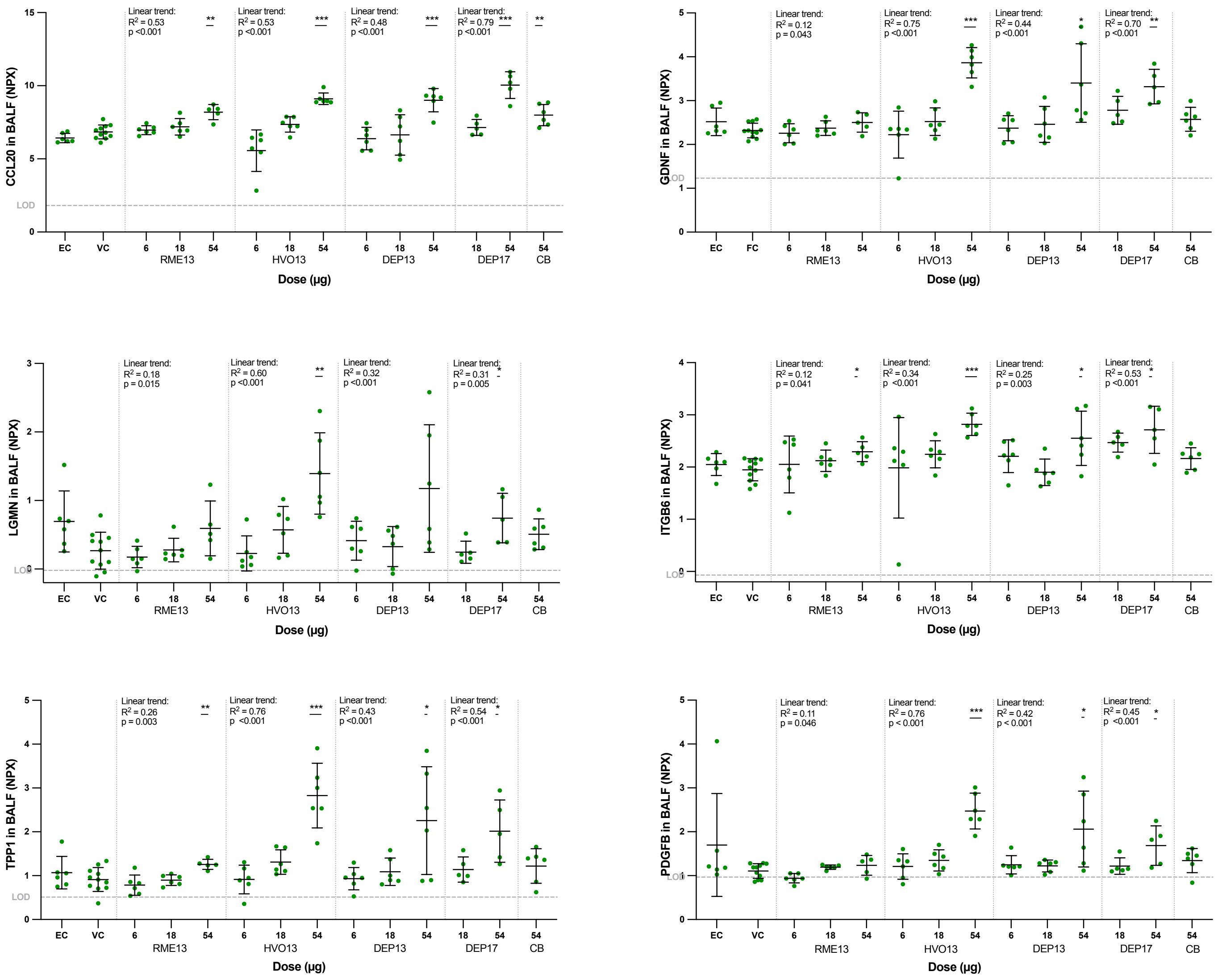

Figure S1. Protein expression in bronchoalveolar lung fluid (BALF) of mice 24 h post instillation exposure to renewable (RME13, HVO13) or petroleum (DEP13, DEP17) diesel exhaust particles. Carbon black (CB) particles were included as reference particles. Protein expression was measured by Proximity Extension Assay (Olink) and expressed as normalized protein expression (NPX) values on a log2-scale. EC, extraction control; VC, vehicle control;

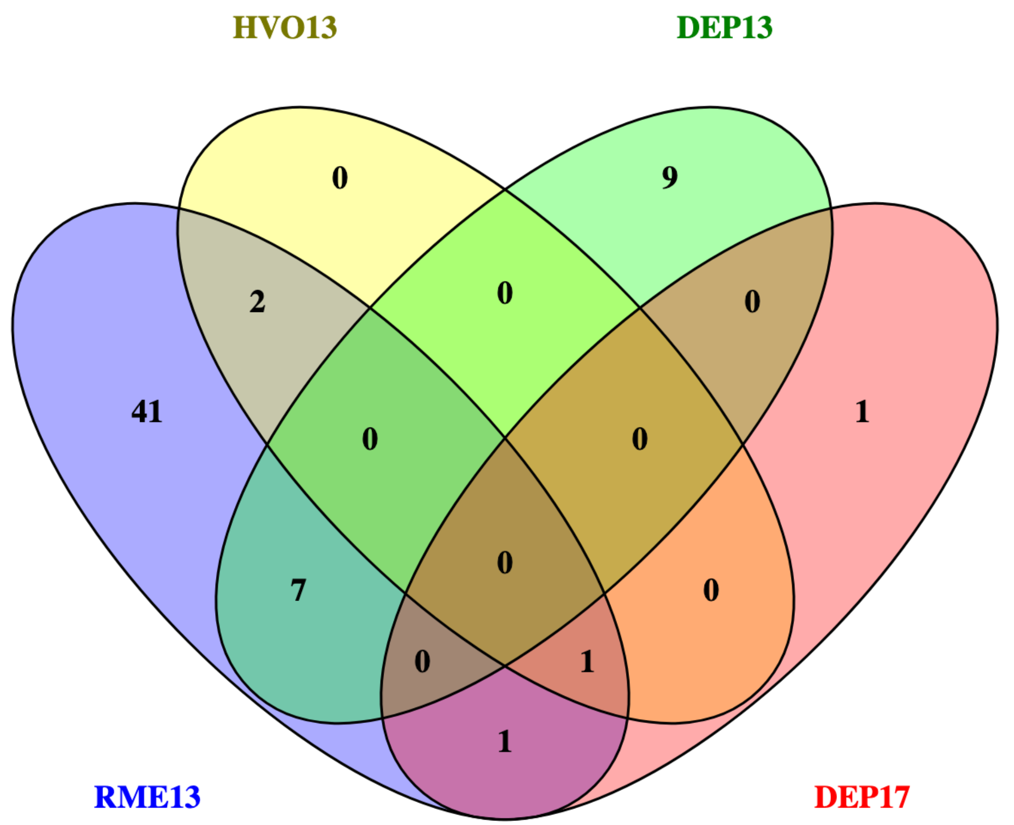

Figure S2. Venn diagram showing the overlap of differentially expressed proteins in plasma in response to exposure to renewable (RME13, HVO13) or petroleum (DEP13, DEP17) diesel exhaust particles on day 1 post-instillation in mice. Protein expression was measured by Proximity Extension Assay (Olink) and differentially expressed proteins were identified based on significant dose-response relationships ($p < 0.05$) in a linear model.

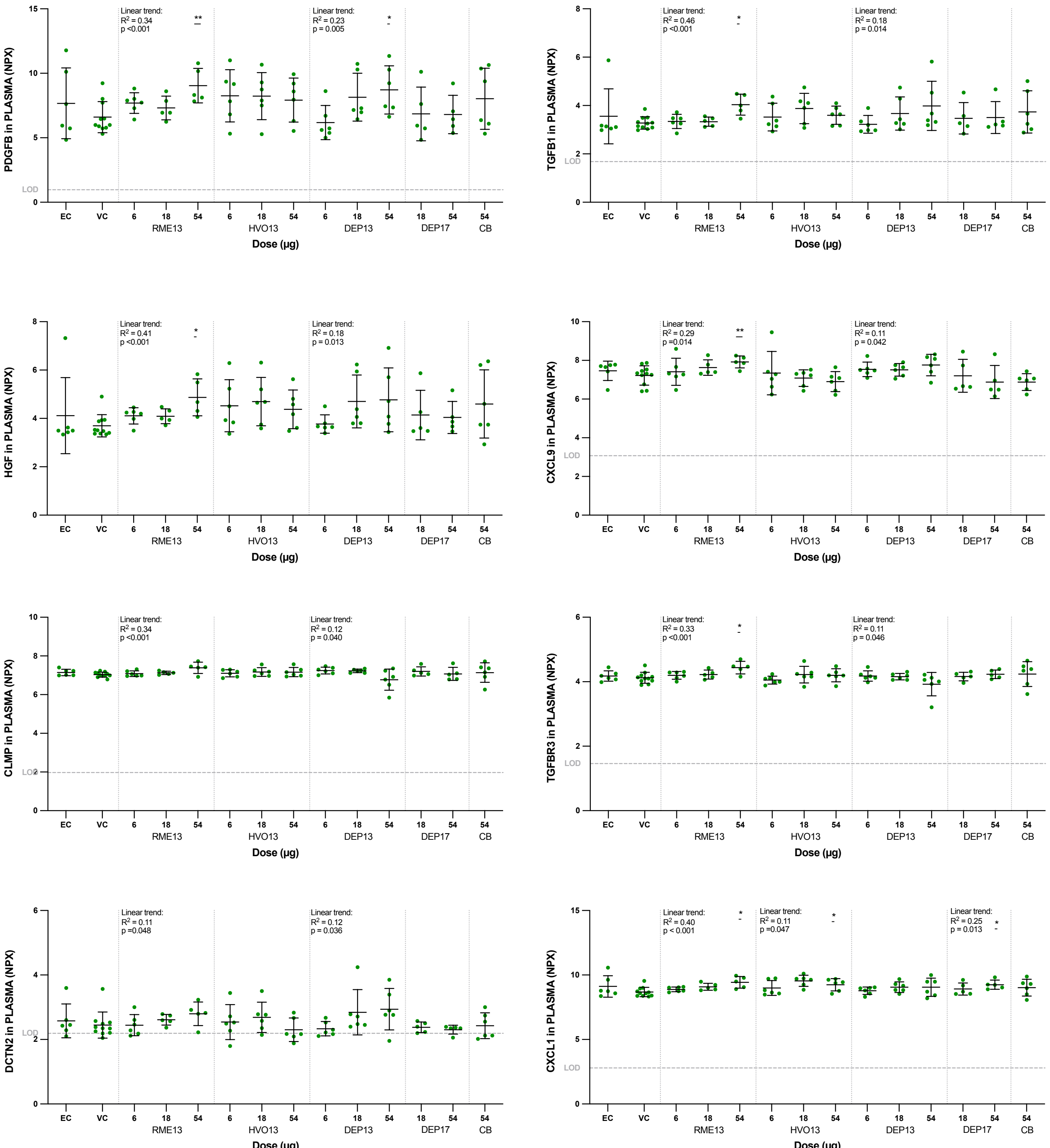

Figure S3. Protein expression in plasma of mice 24 h post instillation exposure to renewable (RME13, HVO13) or petroleum (DEP13, DEP17) diesel exhaust particles. Carbon black (CB) particles were included as reference particles. Protein expression was measured by Proximity Extension Assay (Olink) and expressed as normalized protein expression (NPX) values on a log2-scale. EC, extraction control; VC, vehicle control;

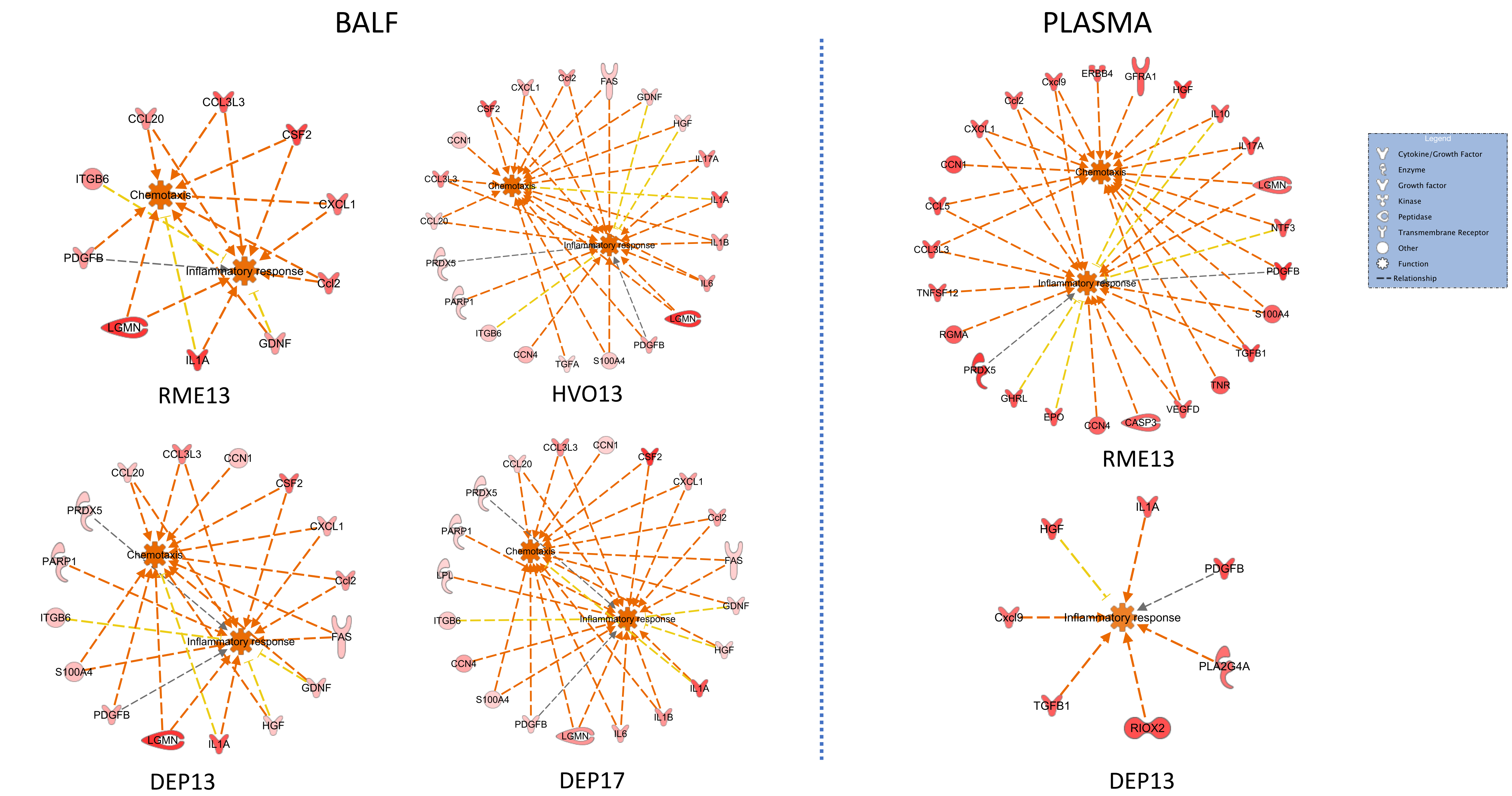

Figure S4. Protein alterations related to the functions "inflammatory response" and "chemotaxis" in bronchoalveolar lung fluid (BALF) or plasma of mice 24 h post instillation exposure to renewable (RME13, HVO13) or petroleum (DEP13, DEP17) diesel exhaust particles. Red boxes indicate positive associations with the exposures, red lines indicate relationships that are consistent with activation of function, yellow lines indicate inconsistent relationships. The colour of the protein symbols are related to the fold change within the respective exposure (highest fold change for each exposure is assigned the brightest colour) and should not be compared between the exposures. Figure generated in PathDesigner, Ingenuity Pathway Analysis.

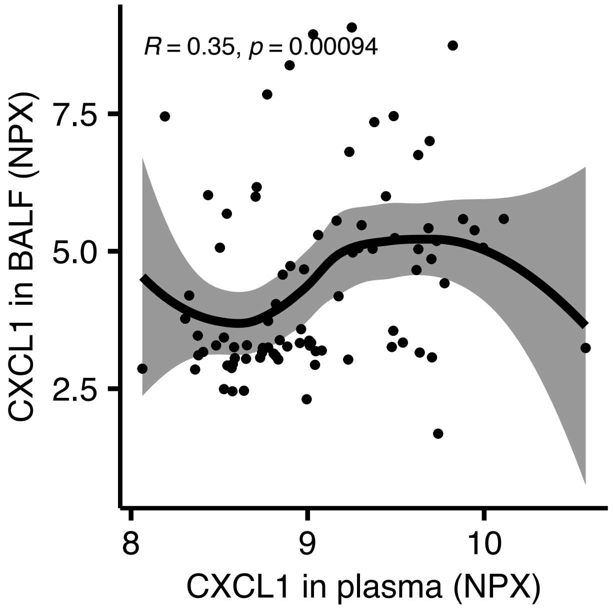

Figure S5. Correlation (spearman) plot between CXCL1 protein expression in bronchoalveolar lung fluid (BALF) and plasma of mice 24 h post instillation exposure to renewable (rapeseed methyl ester, RME13 and hydrogen-treated vegetable oil, HVO13,) and petroleum diesel exhaust particles (DEP13, DEP17). Carbon black (CB) printex particles were included as reference particles. Protein expression was measured by Proximity Extension Assay (Olink) and expressed as normalized protein expression (NPX) values on a log2-scale. Line represents the loess curve and shading stands for the confidence interval. R, spearman correlation coefficient.

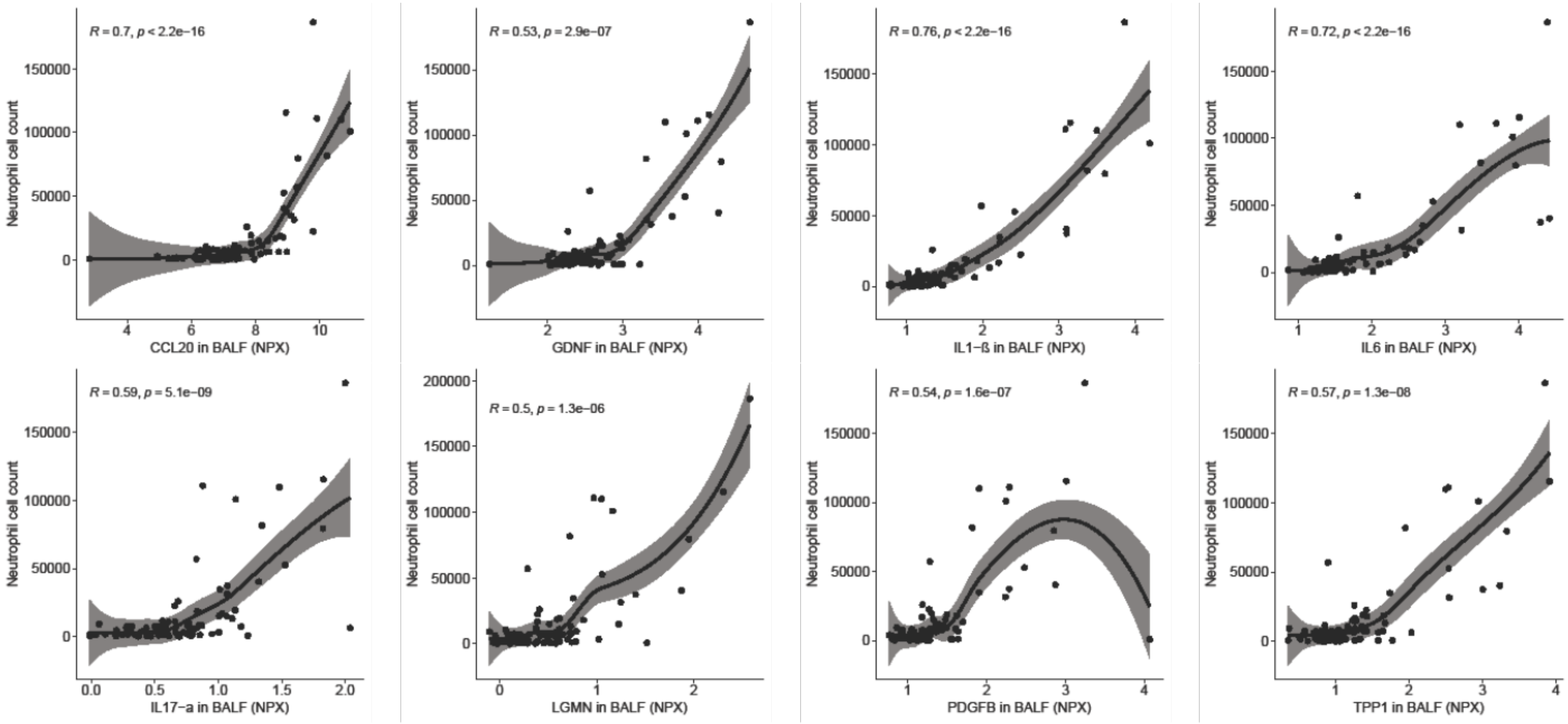

Figure S6. Correlation (Spearman) plots between protein expression in bronchoalveolar lung fluid (BALF) and neutrophil cell count in mice 24 h post instillation exposure to renewable (RME13, HVO13) or petroleum (DEP13, DEP17) diesel exhaust particles . Carbon black (CB) printex particles were included as reference particles. Protein expression was measured by Proximity Extension Assay (Olink) and expressed as normalized protein expression (NPX) values on a log2-scale. Line represents the loess curve and shading stands for the confidence interval. R, spearman correlation coefficient.

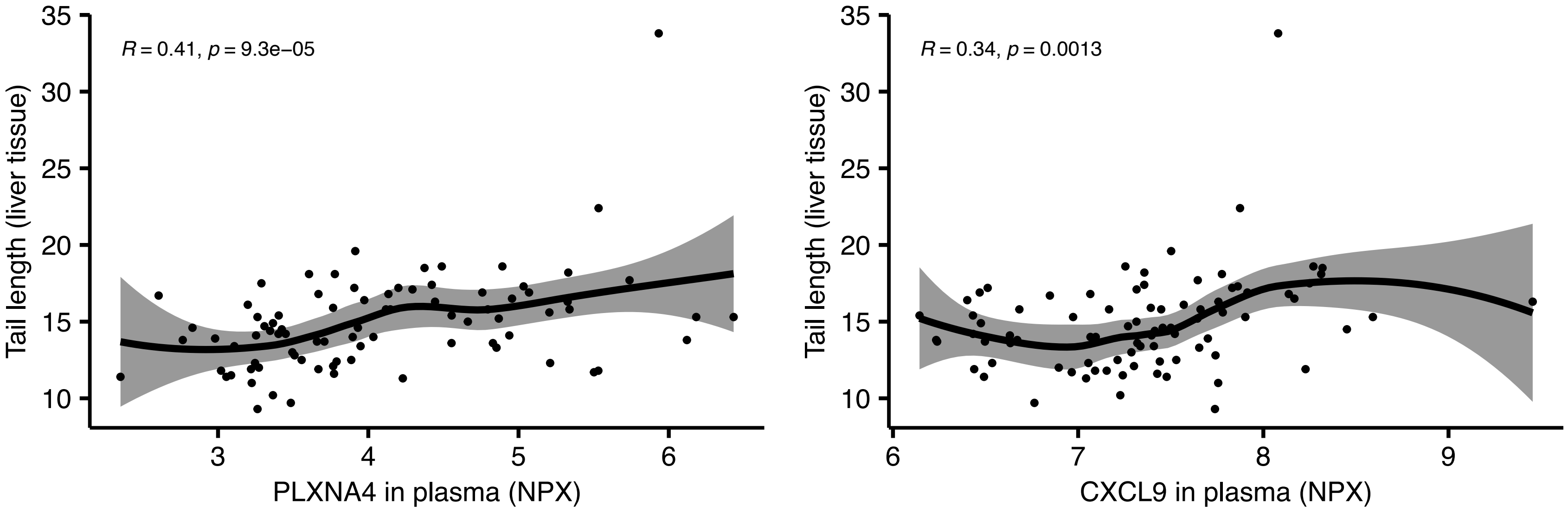

Figure S7. Correlation (spearman) between PLXNA4 and CXCL9 in plasma of mice and tail length in liver tissue 24 h post instillation exposure to renewable (rapeseed methyl ester, RME13 and hydrogen-treated vegetable oil, HVO13,) and petroleum diesel exhaust particles (DEP13, DEP17). Protein expression was measured by Proximity Extension Assay (Olink) and expressed as normalized protein expression (NPX) values on a log2-scale. R, spearman correlation coefficient.
